# Pituitary apoplexy induced by gonadotropin-releasing hormone (GnRH) agonist administration for treatment of prostate cancer: a systematic review

**DOI:** 10.1007/s00432-021-03697-1

**Published:** 2021-06-22

**Authors:** Rishi Raj, Ghada Elshimy, Aasems Jacob, P. V. Akhila Arya, Dileep C. Unnikrishnan, Riccardo Correa, Zin W. Myint

**Affiliations:** 1 Department of Internal Medicine, Division of Endocrinology, Diabetes, and Metabolism, Pikeville Medical Center, Pikeville, KY 41501 USA; 2 Department of Internal Medicine, Division of Endocrinology, Diabetes, and Metabolism, Augusta University, Augusta, GA 39012 USA; 3 Department of Internal Medicine, University of Kentucky, 800 Rose Street, Room No. CC 402, Lexington, KY 40536 USA; 4 Department of Medicine, Government Medical College, Calicut, Kerala 673008 India; 5 Department of Critical Care Medicine, Cloudphysician Healthcare, 7 Bellary Road, Ganganagar, Bengaluru, 560032 India; 6 Division of Endocrinology, Diabetes, and Metabolism, Phoenix VAMC and University of Arizona College of Medicine-Phoenix, Phoenix, AZ 85012 USA; 7 Division of Medical Oncology, Department of Internal Medicine, University of Kentucky, Lexington, KY 40536 USA

**Keywords:** Endocrine system, Pituitary disorders, Pituitary apoplexy, Gonadotropin-releasing hormone (GnRH) agonist, Prostate cancer, Contraindications and precautions, Drugs: endocrine system, Drugs: prostate cancer

## Abstract

**Objective:**

We aimed to review of literature on the clinical presentation, management and outcomes of pituitary apoplexy following gonadotrophic release hormone (GnRH) agonist administration for the treatment of prostate cancer.

**Methods:**

We used PRISMA guidelines for our systematic review and included all English language original articles on pituitary apoplexy following GnRH agonist administration among prostate cancer patients from Jan 1, 1995 to Dec 31, 2020. Data on patient demographics, prostate cancer type, Gleason score at diagnosis, history of pituitary adenoma, clinical presentation, GnRH agonist, interval to pituitary apoplexy, laboratory evaluation at admission, radiologic findings, treatment of pituitary apoplexy, time to surgery if performed, pathology findings, and clinical/hormonal outcomes were collected and analyzed.

**Results:**

Twenty-one patients with pituitary apoplexy met our inclusion criteria. The mean age of patients was 70 (60–83) years. Leuprolide was the most common used GnRH agonist, used in 61.9% of patients. Median duration to symptom onset was 5 h (few minutes to 6 months). Headache was reported by all patients followed by ophthalmoplegia (85.7%) and nausea/vomiting (71.4%). Three patients had blindness at presentation. Only 8 cases reported complete anterior pituitary hormone evaluation on presentation and the most common endocrine abnormality was FSH elevation. Tumor size was described only in 15 cases and the mean tumor size was 26.26 mm (18–48 mm). Suprasellar extension was the most common imaging finding seen in 7 patients. 71.4% of patients underwent pituitary surgery, while 23.8% were managed conservatively. Interval between symptoms onset to pituitary surgery was 7 days (1–90 days). Gonadotroph adenoma was most common histopathologic finding. Clinical resolution was comparable, while endocrine outcomes were variable among patients with conservative vs surgical management.

**Conclusion:**

Although the use of GnRH agonists is relatively safe, it can rarely lead to pituitary apoplexy especially in patients with pre-existing pituitary adenoma. Physicians should be aware of this complication as it can be life threatening. A multidisciplinary team approach is recommended in treating individuals with pituitary apoplexy.

## Introduction

Pituitary apoplexy (PA) is a rare but potentially life-threatening condition of bleeding into the pituitary gland that occurs in 0.6–10% of patients with known pituitary tumors (Nielsen et al. 2006). Only 10–40% of the cases of PA have an identified precipitating factor including the possibilities of angiographic procedures, orthopedic/cardiac surgeries, dynamic testing or medications such as GnRH agonists, anticoagulation therapy and dopamine agonists. Symptoms are broad and range from sudden onset of headache, ocular paresis, reduction in visual fields, and vomiting to altered mental status caused by the rapid enlargement of the pituitary gland due to bleeding and/or infarction usually within a tumor (Nielsen et al. 2006; Fraioli et al. 1990; Bonicki et al. 1993). Gonadotropin-releasing hormone (GnRH) agonists have been used in the management of different conditions including prostate cancer, precocious puberty, uterine bleeding due to endometriosis, as a transgender medicine, and fibroid treatment among others. Androgen deprivation therapy (ADT), consisting of GnRH agonists (leuprolide acetate, goserelin and triptorelin) or GnRH antagonists (degarelix and relugolix), is the treatment foundational treatment for advanced prostate cancer. GnRH agonists work by suppressing luteinizing hormone (LH) production and, therefore, the synthesis of testicular androgens. There have been few reported cases of PA following use of GnRH agonists in patients with prostate cancer. The diagnosis of PA requires a very high index of suspicion. The treatment of PA has remained controversial and was previously considered a neurosurgical emergency (Fraioli et al. 1990; Bonicki et al. 1993) but recent literature suggests the existence of spontaneous clinical recovery, and favors a conservative approach in individualized cases (Rajasekaran et al. 2011; Capatina et al. 2015). The outcomes of pituitary apoplexy are highly variable and can result in a rapid decline in clinical status ranging from subarachnoid hemorrhage or cerebral ischemia, or the patient may have a spontaneous recovery with or without any sequelae. Our study aimed to review the literature of pituitary apoplexy following androgen deprivation therapy for prostate cancer using GnRH agonists and characterize the clinical presentation, management and outcomes. Healthcare professionals should be aware of the association between GnRH agonist use and PA to enable an early recognition and prompt treatment.

## Methods

### Search strategy

**Fig. 1:**
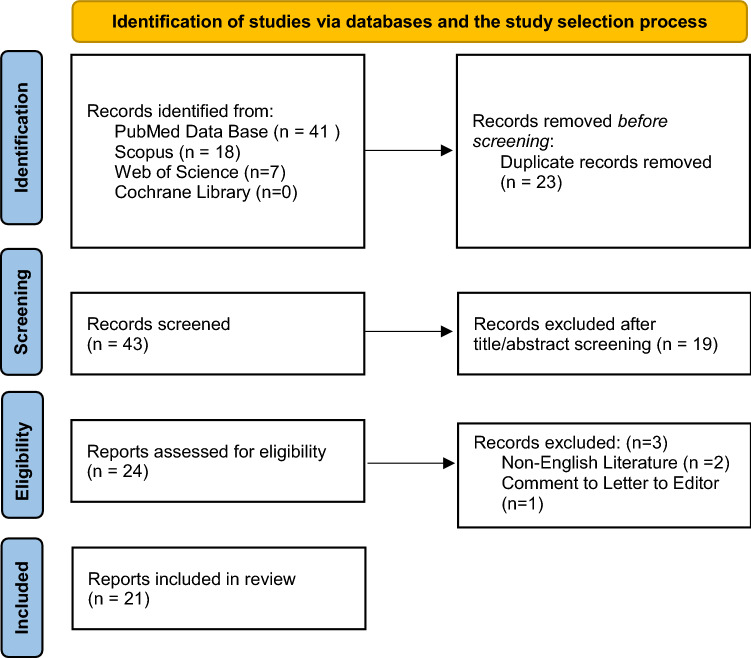
Prisma flow chart for identification of studies via databases and the study selection process

We identified all the peer-reviewed published literature pertaining to pituitary apoplexy following GnRH agonist administration for the treatment of prostate cancer from Jan 1, 1995 to Dec 31, 2020. We adopted Prisma guidelines for our systematic review and used electronic database of MEDLINE, Web of Science, Scopus, and Cochrane Library to identify all the related articles published by Dec 31, 2021. The following keywords were used: “Pituitary Apoplexy”, “Cerebral Hemorrhage”, “Pituitary Diseases”, “Pituitary Neoplasms”, gonadotropin-releasing hormone (GnRH), “Gonadotropin-Releasing Hormone”, “gonadotropin-releasing hormone agonist”, “Prostate Cancer” and “Prostatic Neoplasms”. The identified articles were assessed for inclusion independently by two authors (GE, RR) and divergences were unified through discussion. Eligibility criterion for inclusion in the review was the event of pituitary apoplexy following GnRH agonist administration for the treatment of prostate cancer. Only manuscripts in English language were included in the study. A Prisma flow chart showing the process of identification of articles via databases is shown in Fig. 1.

### Data collection

The information extracted from the selected publications were as follows: author, year, study design, patient demographics, prior history of pituitary adenoma, clinical signs/symptoms at presentation, type of GnRH agonist used, interval to pituitary apoplexy following GnRH agonist use, laboratory evaluation on admission, radiologic findings on presentation, treatment method, time to surgery after presentation, pathology findings, and clinical/hormonal outcome.

### Data analysis

Categorical variables were reported as percentage and continuous variables as means or medians. For the parametric variables, we used *t* test for analysis. For the non-parametric variable, we used Wilcoxon test for analysis. All the statistical analyses were performed using SPSS 26.0. Clinical features and efficacy outcomes were not statistically analyzed further due to small sample size and inconsistency in data reporting.

## Results

### Baseline characteristics (Tables 1 and 2)

**Table 1:** Clinical characteristics of patients diagnosed with pituitary apoplexy following administration of gonadotropin-releasing hormone agonists (GnRH) for treatment of prostate cancer

No.	Author/year	Race/age	Prior pituitary lesion	Drug, dose (mg)	Interval to apoplexy	Clinical features	Treatment/time to surgery	Pathology/IHC	Clinical outcomes	Hormonal outcome
1	Ando (1995) (Letter to editor)	ND/83	ND	Goserelin, 3.6	9 days	H, N/V, VF, F, AMS	Conservative	NA	CR of VF	On replacement steroids. TSH, LH/FSH and GH/IGF1 ND
2	Chanson (1995) (Letter to editor)	ND/78	ND	Triptorelin, 3.75	Few minutes	H, OP	Conservative	NA	CR of OP	Low LH, High FSH. ACTH, TSH, GH/IGF1 ND
3	Morsi (1996)	ND/74	ND	Leuprolide, 2.5	15 min	H, N/V, EP, VA, OP, AMS	Surgery, 3 days	FSH, LH and GH	Died 12-day post-surgery	ND
4	Reznik (1997)	ND/64	ND	Leuprolide, 3.75	4 days	H, OP	Surgery, ND	FSH, LH	ND	ND
5	Eaton (2001)	ND/67	ND	Goserelin, 3.6	4 h	H, N/V, VF, B, AMS	Surgery, 24 h	FSH, LH	PR of VF, PR in B, NR in OP	On replacement steroid/thyroid hormones, LH/FSH and GH/IGF1 ND
6	Spengos (2002)	ND/74	ND	Leuprolide, ND	6 months	H, N/V, OP, M	Surgery Planned	NA	ND	ND
7	Massoud (2006)	C/70	ND	Leuprolide, 11.25	10 days	H, VF, OP	Surgery, 3 months	FSH and LH	PR of VF, PR of OP	ND
8	Blaut (2006)	ND/68	ND	Goserelin, 3.6	4–6 h	H, N/V, OP, AMS	Surgery, ND	GH, ACTH and PRL negative	CR of OP	On replacement steroid/thyroid hormones. LH/FSH low. GH/IGF1 ND
9	Davis (2006)	AA/61	ND	Leuprolide, 30	Few hours	H, N/V, VF, OP, M	Surgery, ND	FSH	CR of VF, CR of OP	ND. Patient did not follow-up post-pituitary surgery
10	Hands (2007)	ND/60	ND	Leuprolide, 22.5	4 h	H, N/V, OP, B, AMS	Surgery, ND	LH	CR of B	On replacement steroid/thyroid hormones. LH, FSH, GH/IGF1 ND
11	Guerra (2010)	AA/60	ND	Leuprolide, ND	Few hours	H, OP	Surgery, ND	LH	ND	ND
12	Sinnadurai (2010)	ND/71	ND	Goserelin, ND	8 weeks	H, N/V, VF, VA, B, OP	Surgery, 4 days	LH, FSH	CR of VF, PR of B	ND
13	Ito (2011) (Letter to editor)	ND/78	ND	Goserelin, 3.6	9 days	H, VF, OP	Surgery, ND	FSH	CR of OP, CR of VF	Normal LH/FSH. ACTH, TSH and GH/IGF1 ND
14	Huang (2013)	A/77	ND	Leuprolide, 3.75	Few hours	H, N/V, VF, VA, OP	Surgery, 10 days	ND	CR of VF, CR of OP	ND
15	Babbo (2014)	AA/60	ND	Leuprolide, ND	Few hours	H, N/V, OP	Surgery, 14 days	LH, FSH	CR of OP	Normal ACTH, TSH, LH/FSH and GH/IGF1
16	Sasagawa (2015)	ND/62	Yes	Leuprolide, 11.25	10 min	H, N/V, OP	Surgery, 20 days	FSH, LH	CR of OP	On replacement steroid and thyroid hormone. Normal LH/FSH and GH/IGF1
17	Guerrero (2015) (Letter to editor)	ND/77	ND	Triptorelin, 22.5	1 h	H, N/V, OP	Surgery, ND	ND	ND	On replacement steroid, Low LH/FSH, TSH and GH/IGF1 ND
18	Keane (2016)	ND/67	Yes	Triptorelin, ND	14 days	H, VF, OP	Surgery, 3 days	FSH, LH	CR of OP	Normal ACTH, TSH, and GH/IGF1. Low LH/FSH
19	Fabiano (2016)	ND/63	ND	Leuprolide, 11.25	3 days	H, N/V, VF, AMS	Conservative	NA	NR of VF	Normal ACTH, TSH, LH, FSH and GH/IGF1
20	Tanios (2017)	ND/85	ND	Leuprolide, 45	4 h	H, N/V, P, EP	Conservative	NA	ND	On replacement steroid. TSH, LH/FSH and GH/IGF1 ND
21	Barbosa (2020)	ND/69	Yes	Leuprolide, 45	Few minutes	H, N/V, OP	Conservative	NA	CR of OP	On replacement steroid, Low LH/FSH, TSH and GH/IGF1 ND

*IHC* immunohistochemistry, *n* number, *C* Caucasian, *A* Asian, *AA* African–American, *ND* not described, *H* headache, *N/V* nausea/vomiting, *OP* ophthalmoplegia, *VF* visual field defect, *VA* visual acuity defect, *P* photophobia, *F* fever, *AMS* altered mental status, *EP* eye pain, *B* blindness, *M* meningism, *ACTH* adrenocorticotrophic hormone, *TSH* thyroid stimulating hormone, *LH* luteinizing hormone, *FSH* follicle secreting hormone, *GH* growth hormone, *PRL* prolactin, *IGF-1* Insulin-like growth factor-1, *CR* complete resolution, *PR* partial resolution, *NR* No resolution

**Table 2:** Baseline characteristics of patients treated with GnRH agonist for prostate cancer

Variables	*n* (% or range)
Age (mean)	70 (60–83)
Race	
African American	3 (14.3%)
Asian	1 (4.8%)
Caucasian	1 (4.8%)
Unknown	16 (79.2%)
Prior history of pituitary adenoma	
Yes	3 (14.3%)
Unknown	18 (85.7%)
No	0
Prostate cancer stage	
Local/locally advanced	8 (38.1%)
Metastatic	4 (19%)
Recurrent	3 (14.3%)
Unknown	6 (28.6%)
Grade group (Gleason score) at diagnosis	
1 (≤ 6)	2 (9.5%)
2 (3 + 4 = 7)	2 (9.5%)
3 (4 + 3 = 7)	0
4 (8)	1 (4.7%)
5 (9,10)	2 (9.5%)
Unknown	14 (66.7%)
Prior prostatectomy	5 (23.8%)
GnRH agonist used	
Leuprolide	13 (61.9%)
Goserelin	5 (23.8%)
Triptorelin	3 (14.3%)
Use of anti-androgen therapy	
Cyproterone acetate	3 (14.3%)
Bicalutamide	2 (9.5%)
Flutamide	1 (4.7%)
Used (drug name not mentioned)	1 (4.7%)
No/unknown	14 (66.7%)

We identified a total of 66 published articles between 1995 and 2020 on database search and 21 cases fulfilled inclusion criteria (Ando et al. 1995; Chanson and Schaison 1995; Morsi et al. 1996; Reznik et al. 1997; Eaton et al. 2001; Spengos et al. 2002; Blaut et al. 2006; Davis et al. 2006; Massoud et al. 2006; Hands et al. 2007; Guerra et al. 2010; Sinnadurai et al. 2010; Ito 2011; Huang et al. 2013; Babbo et al. 2014; Guerrero-Pérez et al. 2015; Sasagawa et al. 2015; Fabiano and George 2016; Keane et al. 2016; Tanios et al. 2017; Barbosa et al. 2020). The mean age of the patients was 70 years. Among the reports which disclosed details about prostate cancer, 38.1% were local or locally advanced prostate cancer and 14.3% were recurrence after prior local treatments. Only 5 cases reported Gleason score and PSA levels at presentation were inconsistently reported. Only 3 patients (14.3%) had a prior history of pituitary adenoma.

Sixty-two (62) % of the patients received leuprolide. The doses of leuprolide associated with pituitary apoplexy varied; three patients used 11.25 mg dose, two were on 3.75 mg, two received 45 mg and one each on 2.5, 22.5, and 30 mg; in three patients the dose of leuprolide was not reported. Among the five patients who received goserelin, four had 3.6 mg subcutaneous dose, while one patient had a subcutaneous implant. One patient each received triptorelin 3.75 and 22.5 mg triptorelin, while in one patient dose was not reported. 7 out of the 21 patients also received prior/concurrent antiandrogen therapy.

### Clinical presentation (Table 3)

**Table 3:** Clinical, laboratory and radiographic presentation at the time of pituitary apoplexy among patients with prostate cancer treated with GnRH agonist

Variable	*N* or median (% or range)			
Interval to apoplexy				
Early Onset (< 24 h)	13 (61.9%)			
Median duration of onset	13.4 h (5 min–6 h)			
Late onset (> 24 h)	8 (38.09%)			
Median duration of onset	8.9 days (3 days–6 months)			
Clinical presentation				
Headache	21 (100%)			
3rd/4th/6th cranial nerve palsy	18 (85.7%)			
Nausea/vomiting	15 (71.4%)			
Visual field defect	9 (42.9%)			
Altered mental status	6 (28.6%)			
Visual acuity defect	3 (14.3%)			
Blindness	3 (14.3%)			
Eye pain	2 (9.6%)			
Meningism	2 (9.6%)			
Fever	1 (4.8%)			
Photophobia	1 (4.8%)			
Central Hormone levels	Normal	Deficient	Elevated	Unknown
Prolactin	7	5	2	7
ACTH	5	3	0	13
LH	8	4	4	5
FSH	6	4	6	5
TSH	7	6	0	8
GH/IGF-1	6	4	0	11
Imaging findings^a^				
Mean maximum dimension Mean volume Suprasellar extension Cavernous sinus extension Infrasellar extension	26.3 mm (18–48 mm) 9600 mm^3^ (4000–50,400 mm^3^) 11 (52.4%) 9 (42.9%) 1 (4.8%)			

^*a*^Tumor size was described in only 15 out of 21 cases

Thirteen (61.9%) patients developed symptoms within 24 h of administration of GnRH agonist, while eight patients (38.09%) developed symptoms between 1 day and 6 months. Median duration to onset of symptoms or diagnosis of apoplexy post-GnRH administration was 5 h. Four patients who were on prior/concurrent anti-androgen therapy had early onset PA.

All patients had headache as the initial presenting symptom. Twenty patients (95.2%) had visual symptoms at presentation with ophthalmoplegia present among 18 and visual field defect in 9 patients. Three patients had blindness at presentation.

### Laboratory findings at presentation (Table 3, Fig. 2)

**Fig. 2:**
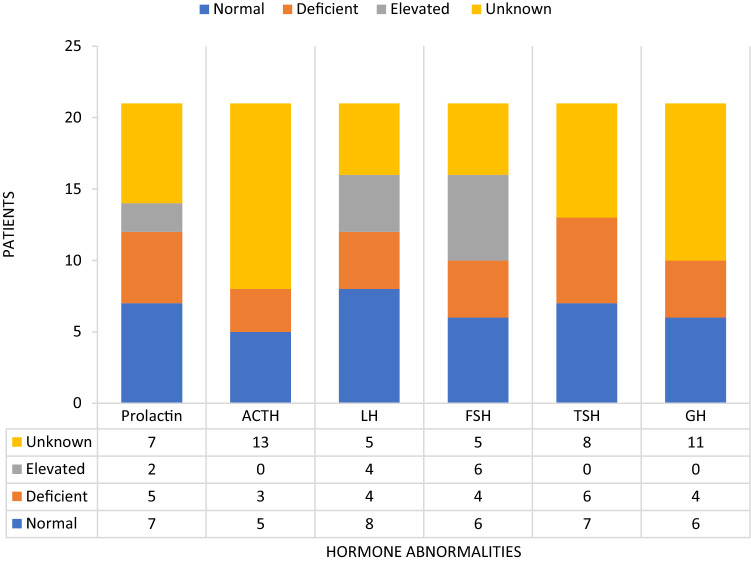
Endocrine abnormalities at presentation reported among the study patients

Only 8 patients had reported complete anterior pituitary hormone evaluation and 16 cases had at least one pituitary hormone evaluation reported. The most common endocrine abnormality on presentation was follicle-stimulating hormone (FSH) elevation (*n* = 6) followed by thyroid-stimulating hormone (TSH) deficiency (*n* = 6) and low prolactin levels (*n* = 5). One patient had hyponatremia secondary to Syndrome of Inappropriate Anti Diuretic Hormone (SIADH) and another patient had central diabetes insipidus.

### Imaging findings (Table 3)

Tumor size was described in only 15 cases. The mean maximum dimension was 26.26 mm (range: 18–48 mm). Nine cases described the tumor volume, with a mean volume of 9600 mm^3^ (range: 4000–50,400 mm^3^). Seven patients had suprasellar extension, 5 with cavernous sinus extension and 4 had both suprasellar and cavernous sinus extension. Infrasellar extension was rare with only one reported case.

### Management

Among 21 patients, 15 (71.4%) underwent pituitary surgery, while 5 (23.8%) were conservatively managed. One patient had surgery planned but not reported as completed. Among patients managed with pituitary surgery, the interval between symptoms onset to pituitary surgery was approximately 7 days (range: 1–90 days).

### Pathology

Pathologic findings were described among 12 out of the 15 patients who underwent surgical treatment for pituitary apoplexy. 10 tissue samples were LH and FSH positivity by immunohistochemistry, while one was GH positive. Two tissue samples were inconclusive on analysis with hematoma and necrotic tissue identified.

### Outcomes (Tables 4 and 5)

**Table 4:** Reported clinical outcomes after surgical and conservative management of pituitary apoplexy

Clinical Outcomes	Post-surgical management (*n* = 15, 71.4%)				Post-conservative management (*n* = 5, 23.8%)			
	CR	PR	NR	ND	CR	PR	NR	ND
Visual field defect	4	2	0	9	1	0	1	3
Blindness	1	2	0	12	0	0	0	5
Ophthalmoplegia	7	1	0	7	2	0	0	3

*CR* Complete resolution, *PR* partial resolution, *NR* No resolution, *ND* not described

Surgery was planned in article by (Spengos et al. 2002), and hence was not included in outcome analysis

**Table 5:** Reported hormonal outcomes after surgical and conservative management of pituitary apoplexy

Hormonal outcomes	Post-surgical management (*n* = 15, 71.4%)			Post-conservative management (*n* = 5, 23.8%)		
	Def	No Def	Unknown	Def	No Def	Unknown
ACTH	5	2	8	3	1	1
TSH	4	2	9	0	1	4
LH/FSH	3	3	9	2^a^	1	2
GH/IGF1	0	3	12	0	1	4

*Def* Deficiency, *ACTH* adrenocorticotrophic hormone, *TSH* thyroid stimulating hormone, *LH* luteinizing hormone, *FSH* follicle secreting hormone, *GH* growth hormone, *IGF-1* Insulin-like growth factor-1

^a^In one patient, LH was deficient; however, FSH remained high post-conservative management

All patient who underwent pituitary surgery had complete or partial resolution of symptoms. Conservative management resulted in complete resolution of symptoms in 3 patients, while 1 patient had a persistent visual field defect. Endocrine outcomes were variable for surgical as well as conservative management. Patients with hormonal abnormalities at presentation continued to have persistent abnormalities post-management, whether surgical or conservative. Two patients who underwent surgical management died. One patient died of cardiac arrest 12-day post-surgery and another patient died of complications from malignant melanoma 8 months later (Morsi et al. 1996; Eaton et al. 2001).

### Post-PA prostate cancer treatment

Treatment of prostate cancer after PA was not described in 11 patients. Among the five patients treated conservatively, one patient received radiation treatment for prostate cancer following PA and the management of prostate cancer was not described in the rest (Barbosa et al. 2020).

Among 15 patients treated with surgery, 7 did not describe further management of prostate cancer. One patient with locally advanced prostate cancer had a normal testosterone level after surgery and was on observation for prostate cancer with stable prostate specific antigen (PSA) levels after 1 year (Massoud et al. 2006). GnRH agonist rechallenge was attempted in 4 patients. As reported by Reznik et al., leuprolide rechallenge failed to suppress LH/FSH and testosterone. This patient was treated with flutamide and underwent orchiectomy (Reznik et al. 1997). The other 3 patients had successful rechallenge with GnRH agonists (Blaut et al. 2006; Davis et al. 2006; Sasagawa et al. 2015).

Two patients received androgen receptor blocker—bicalutamide only (Sinnadurai et al. 2010; Ito 2011), while one patient had radiation treatment in addition to bicalutamide (Babbo et al. 2014). Degarelix and radiation was used for prostate cancer management in the report from Keane et al. (2016).

## Discussion

Our review focused on patients receiving a GnRH agonist as part of ADT for treatment of prostate cancer who developed PA. The mean age of patients included in the series corresponds to the peak age of prostate cancer diagnosis, which is between 65 and 74 years (National Cancer Institute’s Surveillance, Epidemiology, and End Results program 13). PA was reported with 3 of the currently FDA approved GnRH agonists—goserelin, leuprolide and triptorelin—and there were no available reports of associated PA with buserelin or histrelin.

A history of pituitary adenoma prior to presentation was reported only in 3 cases. However, previously undiagnosed adenomas could have been present in additional cases considering the estimated prevalence of pituitary adenoma to be 77.6 per 100,000 in general population (Fernandez et al. 2010). Although early presentation (within 24 h) was more common, unusually late presentation at 8 weeks and 6 months was also observed (Spengos et al. 2002; Sinnadurai et al. 2010). Clinical presentation was variable. Headache was the most frequent and the earliest of symptoms, which occurred in all of the patients described. This symptom could be explained by the stretching and stimulation of the hypophyseal capsule, with increased intracranial pressure and/or hemorrhage into the subarachnoid space (Li et al. 2020). Cranial nerve palsies and systemic symptoms such as nausea and vomiting were also common.

Though limited in number of case reports have suggested a causative role of GnRH agonists in development of PA, but the exact pathophysiology is not well established. When administered, GnRH agonists binds to GnRH receptors on pituitary gonadotropin-secreting cells and stimulate gonadotropin secretion: FSH and LH. The GnRH agonists are more potent and have a longer half-life than native GnRH. With the initiation of GnRH agonist treatment there is a dramatic and transient surge of LH and FSH before the levels fall. This LH flare, might, in addition, correlate to transient prostate cancer growth as well as with an increase in size of occult pituitary tumors (Rajasekaran et al. 2011; Capatina et al. 2015). The unique rich vasculature of the pituitary gland, combined with the fragility of the tumor blood vessels can further exacerbate the hemorrhagic tendency. The expanding pituitary tumor, which results from the hemorrhage in the adenoma leads to the compression of the infundibular and hypophyseal vessels, which can ultimately results in pituitary infarction (Blaut et al. 2006; Li et al. 2020; Abbott and Kirkby 2004; Briet et al. 2015; Powell et al. 1974). Another study suggested that pharmacological agents may stimulate the growth of the pituitary adenoma and also can have a direct impact on a tumor’s microvasculature leading to vasoconstriction with subsequent impairment of oxygen and nutritional supply of pituitary adenomas (Okuda et al. 1994). Gonadotroph adenomas, the majority of which are non-functioning adenomas, are the most common adenomas associated with the occurrence of PA. This is consistent with our review, where the majority of PA were found to be gonadotroph adenomas on histopathological examination and were silent pituitary adenoma until presentation as PA.

Endocrine hormone evaluation at the onset of PA was mentioned in most cases, however, was incomplete especially regarding values of ACTH and GH/IGF1. Many of the cases also had missing information on pre- and post-treatment for prolactin, TSH, and FSH/LH. Given the patient’s age group, we would expect some degree of elevation in the gonadotropin hormones. Despite this, FSH elevation was present in only 6 cases. Gonadotropin adenoma was also the most common finding on pathology (silent or non-silent). Although the majority of patient’s underwent pituitary surgery, the factors determining choice of surgical vs conservative management were unclear. Irrespective of management choice, most patients had partial or complete resolution of symptoms. The development of pituitary hormone deficiency is an expected outcome of surgical management, but was also present in the conservatively managed patients.

### Author’s recommendations

Prostate cancer will rarely metastasize to the brain and brain imaging is not routinely done for these patients. A screening pituitary MRI before the use of GnRH agonist is not cost effective, but we suggest a focused review of the pituitary gland from prior radiological images if available. In Babbo et al.’s report, a patient with prior pituitary microadenoma who underwent ADT did not develop PA which suggests that not all patients with pituitary adenoma will develop PA with ADT (Babbo et al. 2014). Hence, the presence of a pituitary adenoma should not be considered as an absolute contraindication for ADT. In these patients as well as those who are at higher risk of PA with GnRH agonist use, GnRH antagonists such as degarelix and relugolix may be considered. The role of concurrent use of antiandrogen therapies such as bicalutamide, flutamide, or cyproterone acetate in this scenario is not clear. As bicalutamide and flutamide only have peripheral activity due to poor penetration of blood brain barrier, their use may have minimal to no effect on pituitary gland (Furr 1996; Sardanons 1989). On the other hand, cyproterone acetate (not currently FDA approved in United States for use in patients with prostate cancer) is known to cross blood–brain barrier (Denmeade and Isaacs 2002). Cyproterone acetate is also known to worsen prolactinoma and hence has the potential to result in PA among patients with occult or known pituitary adenoma (Raj et al. 2018). In our review, 3 patients with prior/concurrent use of cyproterone acetate developed PA.

Patients who develop visual symptoms, headache and nausea or vomiting after initiation of ADT should promptly undergo a pituitary hormonal workup and brain imaging studies for the diagnosis and early appropriate management of PA. The course of PA is variable and management should be individualized. A thorough clinical evaluation by a multidisciplinary team of specialists including neurosurgeons, endocrinologists, neurologist and oncologist is required, especially in patients with evidence of pituitary macroadenomas prior to the start of these agents. The aim of the therapy is to improve the patient’s symptoms and relieve the compression of the surrounding structures. This can be achieved rapidly by surgical intervention while being mindful of the risks of surgical complications. Other options include conservative management with high dose steroids, especially in patients with corticotropic deficiency which is frequent (Briet et al. 2015). A discussion of risks and benefits with the patient is recommended prior to choosing the management route. The available case reports do not mention treatment-related complications or long-term outcomes of these patients. We have summarized key elements in diagnosis and management of PA in patients treated with GnRH agonists in Table 6.

**Table 6:** Key points in diagnosis, management and follow-up of pituitary apoplexy in patients with prostate cancer treated with GnRH agonist therapy^a^

1. Suspected pituitary apoplexy (acute severe headache), perform thorough history and physical and look for neuro-ophthalmic symptoms
2. Assess vital signs and fluid status. Urgent biochemical assessment with full blood count, coagulation studies (requirement for surgical management), urea and electrolytes (hyponatremia in 40% patients), liver function test and detailed endocrine assessment (IGF1, GH, Prolactin, TSH, Free T4, LH, FSH, ACTH, cortisol, and testosterone; as baseline and to rule out hypopituitarism, which occurs in 50–80% patients.) If possible, laboratory work up should be done prior to administration of steroids.)
3. Urgent steroid and fluid replacement should be considered for hemodynamic instability
4. Urgent MRI or dedicated pituitary CT if MRI is contraindicated to confirm diagnosis
5. Evaluation by multidisciplinary team including endocrinologist, ophthalmologist and neurosurgeons after confirmation of diagnosis. Consideration of surgical vs conservative management based on clinical status, patient and physician decision
6. Rechallenge of ADT with GnRH analogues not recommended in conservatively managed patients. May consider GnRH antagonists or orchiectomy
7. Follow-up endocrine evaluation and formal ophthalmologic evaluation should be considered at 4–8 weeks following PA. Long-term follow-up with annual biochemical assessment of pituitary function should also be considered

^a^Adapted from UK guidelines for the management of pituitary apoplexy (Rajasekaran et al. 2011)

There are limitations for this retrospective review of the literature. The case reports lack uniformity and completeness in describing details of laboratory evaluation and management. The language was restricted to English leading to the exclusion of few case reports. In addition, with very few reported cases in the literature and short follow-up, pursuing a full outcome analysis is not possible.

## Conclusion

Use of GnRH agonist in patients with prostate cancer can lead to endocrine complications including PA. Physicians should be aware of this complication as it is considered life threatening and carefully observe the patients during administration of these pharmacological agents. In addition, they should inform the patients regarding warning signs and symptoms that may occur with time, especially in the setting of a prior pituitary adenoma. A careful evaluation by a multidisciplinary team including endocrinologist, oncologist and neurosurgeons is needed if PA occurs; an understanding of the risk factors can aid in an early diagnosis and the management of these patients.

## Data Availability

The data that support the findings of this study are available from the corresponding author upon request. The authors declare that all data supporting the findings of this study are available within the paper.

## Abbreviations

n: Number
C: Caucasian
A: Asian
AA: African–American
ND: Not described
H: Headache
N/V: Nausea/vomiting
OP: Ophthalmoplegia
VF: Visual field defect
VA: Visual acuity defect
P: Photophobia
F: Fever
AMS: Altered mental status
EP: Eye pain
B: Blindness
M: Meningism
ACTH: Adrenocorticotrophic hormone
TSH: Thyroid stimulating hormone
LH: Luteinizing hormone
FSH: Follicle secreting hormone
GH: Growth hormone
IGF-1: Insulin-like growth factor-1
CR: Complete resolution
PR: Partial resolution
NR: No resolution
